# Invasive ventilation and mortality in critically ill nonagenarians: a retrospective cohort study

**DOI:** 10.1186/s40001-026-03928-6

**Published:** 2026-01-28

**Authors:** Markus Haar, Fabian Gleibs, Anna Carola Hertrich, Jakob Müller, Rikus Daniels, Pauline Theile, Stefan Kluge, Kevin Roedl

**Affiliations:** 1 Department of Intensive Care Medicine, University Medical Center Hamburg-Eppendorf, Martinistr. 52, 20251 Hamburg, Germany; 2 Department of Anaesthesiology, Tabea Hospital Hamburg, Hamburg, Germany

**Keywords:** Nonagenarians, Invasive ventilation, Intensive care, Survival analysis, Elderly, Critical illness

## Abstract

**Background:**

ICU admissions among very old patients are increasing. Invasive ventilation (IV) is common, but its benefit in patients aged ≥ 90 years is uncertain given high mortality and ethical concerns.

**Methods:**

This retrospective cohort study analysed all consecutive ICU patients aged ≥ 90 years admitted between 2008 and 2023 at a tertiary care centre in Germany. Demographic, clinical, and outcome data were extracted from electronic health records. Multivariable logistic regression was used to identify predictors of hospital mortality, while Kaplan–Meier survival analysis and Cox proportional hazards regression were used to assess predictors of 1-year mortality.

**Results:**

Of 113,950 patients, 1422 (1.25%) aged ≥ 90 years were identified (median 92 years, IQR 91–94; 66% female). IV was administered to 434 patients (31%), while 988 (69%) were not invasively ventilated. Median ICU length of stay was 1.7 days (IQR 1–4) overall. Among ventilated patients, the median duration of IV was 13 h (IQR 4–44), and 66% received IV for less than 24 h. IV was associated with higher illness severity at admission (SOFA score 9 [IQR 7–11] vs. 2 [IQR 1–4], p < 0.001), longer ICU stays (2.9 days [IQR 1.1–7.4] vs. 1.5 days [IQR 0.9–3.1], p < 0.001), as well as higher requirement of vasopressors at admission (0.112 µg/kg/min [IQR 0.056–0.278] vs. 0.072 µg/kg/min [IQR 0.038–0.133], p < 0.001) and renal replacement therapy (3.5% [n = 15] vs. 1.7% [n = 17], p = 0.042). In patients requiring IV, ICU and hospital mortality were 35.7% (n = 155) and 49.3% (n = 214) vs. 11.5% (n = 114) and 21.5% (n = 212) in non-IV patients, respectively (both p < 0.001). Independent and significant predictors of hospital mortality included prolonged IV duration (> 72 h: OR 4.01), peak lactate ≥ 4 mmol/l within 72 h (OR 6.67), as well as elevated SOFA scores (4–7: OR 1.96, ≥ 8: OR 3.46), and CCI ≥ 3 (OR 1.74) at admission. One-year mortality risks were 73.2% (95% CI 68.5–77.3%) and 53.4% (95% CI 50.0–56.6%) for IV and no-IV patients (p < 0.001), respectively.

**Conclusions:**

In this selected cohort of ICU patients aged ≥ 90 years, invasive ventilation–particularly beyond 72 h–identified a subgroup with very high ICU and hospital mortality and greater illness severity. These observational data support using invasive ventilation in nonagenarians as a trigger for early, patient-centred goals-of-care discussions, rather than as evidence that ventilation itself causes excess mortality.

**Supplementary Information:**

The online version contains supplementary material available at 10.1186/s40001-026-03928-6.

## Background

As a consequence of low fertility rates and increased life expectancy the European population has been ageing for decades and will continue to do so [1]. During the period between 2019 and 2050, the number of European Union citizens aged between 75 and 84 years is projected to expand by 56.1%, while those aged 85 years and older will more than double (113.9%) [2]. This holds true for Germany, where the current proportion of nonagenarians and centenarians (≥ 90 years) currently accounts for 1.04% (882 thousand) and is expected to surpass 1.54% (1.30 million) by 2040, and 2.63% (2.17 million) by 2070 (DESTATIS model: BEV-VARIANTE-02, G2L2M2) [3]. Due to the positive association between age and multimorbidity this trend will pose a considerable burden for healthcare [4, 5]. Furthermore, acute and chronic illnesses in this age group often lead to treatment in intensive care units (ICUs) [6–9], yet the decision to provide invasive therapies in patients of advanced age remains ethically complex and challenging for clinicians [10]. Moreover, frailty is associated with worse prognosis in the ICU context [11–13].

Invasive ventilation (IV) is a potentially life-saving intervention frequently employed in ICUs [14, 15]. However, in nonagenarians and centenarians, the appropriateness of this intervention can be controversial due to concerns about the balance between potential benefits, quality of life, and the risk of adverse outcomes [1, 5, 7, 9, 14–16]. The prognosis of elderly patients requiring IV is generally poor, with high rates of mortality [16, 17] and prolonged hospital stays, raising questions about the ethical justification for aggressive treatment in this vulnerable population. Survivors of ARDS and other forms of critical illness frequently experience long-term physical, cognitive, and psychological impairments with reduced health-related quality of life, underscoring the importance of patient-centred outcomes when considering invasive treatments in very old patients [18–21]. Despite the increasing number of elderly ICU admissions, data on the outcomes of IV in patients aged 90 years and above is relatively scarce.

Understanding the clinical trajectory, survival outcomes, and associated complications of IV in this demographic is crucial for informed treatment decisions and improving care strategies. This retrospective study aims to address this gap by examining the outcomes of nonagenarians and older ICU patients who received IV.

## Methods

### Cohort

Data from all patients aged 90 years and older who were consecutively admitted to the Department of Intensive Care Medicine at the University Medical Centre Hamburg-Eppendorf (Germany) between January 2008 and June 2023 were retrospectively analysed. The department comprises 10 intensive care units (ICUs) with a total capacity of more than 100 beds, providing care for critically ill adult patients.

Decisions regarding ICU admission and initiation of IV are made by the attending intensivist in consultation with the referring teams and, where possible, the patient and family. There are no formal age cut-offs for ICU admission or IV; instead, decisions are based on acute illness severity, comorbidities, presumed prognosis, and the anticipated balance between potential benefit and treatment burden. The very old patients included in this study therefore represent those in whom ICU admission and invasive treatment were deemed potentially appropriate.

Patients were included if they were aged 90 years or older at the time of ICU admission; those younger than 90 years were excluded from the analysis.

### Data extraction and preparation

Data were extracted from the hospital’s electronic health record system (Integrated Care Manager^®^ [ICM], Version 9.1–Draeger Medical, Luebeck, Germany). Extracted variables included demographic information (age, sex, weight, height), admission category (non-surgical, elective surgical, or emergency surgical), comorbidities, length of ICU and hospital stay, clinical outcomes, frequency of organ support interventions (including IV, renal replacement therapy [RRT], and vasopressor use), and routine laboratory parameters. Illness severity at ICU admission was quantified using the Sequential Organ Failure Assessment (SOFA) score and the Simplified Acute Physiology Score II (SAPS II), which are routinely collected in our department [22, 23]. The Charlson Comorbidity Index (CCI) was used to assess the burden of chronic comorbid conditions [24]. Validated measures of frailty, pre-ICU functional status, cognitive impairment, and health-related quality of life were not routinely available in the electronic health record and could therefore not be included in this analysis.

### Statistical analysis

Data preparation and cleaning were performed using Python (Version 3.11) and Pandas. Statistical analyses were conducted in R (Version 4.4.1). Categorical variables were compared using Chi-square or Fisher’s exact tests as appropriate, while continuous variables were reported as median and interquartile range (IQR) and compared using the Mann–Whitney-U test. A two-sided *p*-value of < 0.05 was considered statistically significant.

To address the irregular timing of clinical measurements, time-weighted averages of laboratory and blood gas parameters were calculated for the first six hours following ICU admission and were used to describe admission characteristics and inform multivariable analyses.

For hospital mortality, multivariable logistic regression models were constructed to identify independent predictors of death at hospital discharge. Age, sex, admission category (non-surgical, elective surgical, emergency surgical), CCI, admission SOFA score, admission SAPS II, peak lactate within 72 h, haemoglobin, and IV duration category (no IV, < 24 h, 24–72 h, > 72 h) were entered as covariates to account for comorbidity and baseline illness severity. Results were presented as odds ratios (ORs) with 95% confidence intervals (CIs).

Survival analysis was performed for patients who were discharged alive from the hospital, using the Kaplan–Meier method. Post-discharge mortality data were obtained from the federal population registry. Survival was stratified by IV status and IV duration (no IV, < 24 h, 24–72 h, > 72 h). Cumulative mortality at 30, 60, and 90 days, and at 6 and 12 months, was summarised using model-derived estimates. For long-term mortality among hospital survivors, a Cox proportional hazards model was fitted using the maximum available follow-up to evaluate associations between clinical variables and mortality. Age, admission category, CCI, peak lactate, haemoglobin, and IV status/duration were entered as covariates, and results are reported as hazard ratios (HRs) with 95% CIs.

A Sankey diagram was constructed to visualise the distribution of IV duration categories (no IV, < 24 h, 24–72 h, > 72 h) against 1-year survival status (alive vs. deceased). Core variables (age, sex, invasive ventilation status, ICU and hospital mortality) were complete. Admission category and other covariates had only small amounts of missing data, and analyses were based on complete cases for each model; no multiple imputation was performed.

The study adhered to the STrengthening the Reporting of OBservational studies in Epidemiology (STROBE) guidelines [25].

## Results

### Baseline characteristics

Of 113,950 patients within the observation period, a total of 1,422 (1.25%) aged 90 years or older were included in the analysis. The median age was 92 years (IQR 91–94), and 66% (n = 936) were female. IV was administered to 434 patients (31%). Median BMI was 23.4 kg/m^2^ (IQR 21.0–25.9), and most patients were admitted for either non-surgical (35%) or elective surgical (35%) admission causes. Illness severity at ICU admission was moderate overall, with a median SOFA score of 4 and SAPS-II of 36. The median ICU length of stay was 1.7 days (IQR 1.0–4.0). Comorbidity burden, as measured by the CCI, was moderate (2, IQR 1–3). ICU mortality was 19%, and hospital mortality was 30%. Baseline characteristics (Table 1) were broadly similar between IV and no-IV groups with respect to age, sex, BMI. However, patients receiving IV were less frequently female (60% vs. 68%, p = 0.003), and were more commonly admitted for emergency surgery (32% vs. 29%) and non-surgical indication (38% vs. 34%; p = 0.015). Median SOFA scores were higher in IV patients (9 [IQR 7–11] vs. 2 [IQR 1–4], p < 0.001), as were SAPS-II scores (39 [IQR 32–48] vs. 35 [29–41], p < 0.001). In the IV group RRT was required more frequently (3.5% vs. 1.7%, p = 0.042), ICU length of stay was longer (2.9 days [IQR 1.1–7.4] vs. 1.5 days [IQR 0.9–3.1], p < 0.001), and admission norepinephrine as well as lactate levels were significantly elevated (norepinephrine: 0.112 µg/kg/min [IQR 0.056–0.278] vs. 0.072 [IQR 0.038–0.133] µg/kg/min; lactate: 1.44 mmol/l [IQR 0.90–2.57] vs. 1.01 mmol/l [IQR 0.70–1.60]; both p < 0.001).

**Table 1:**
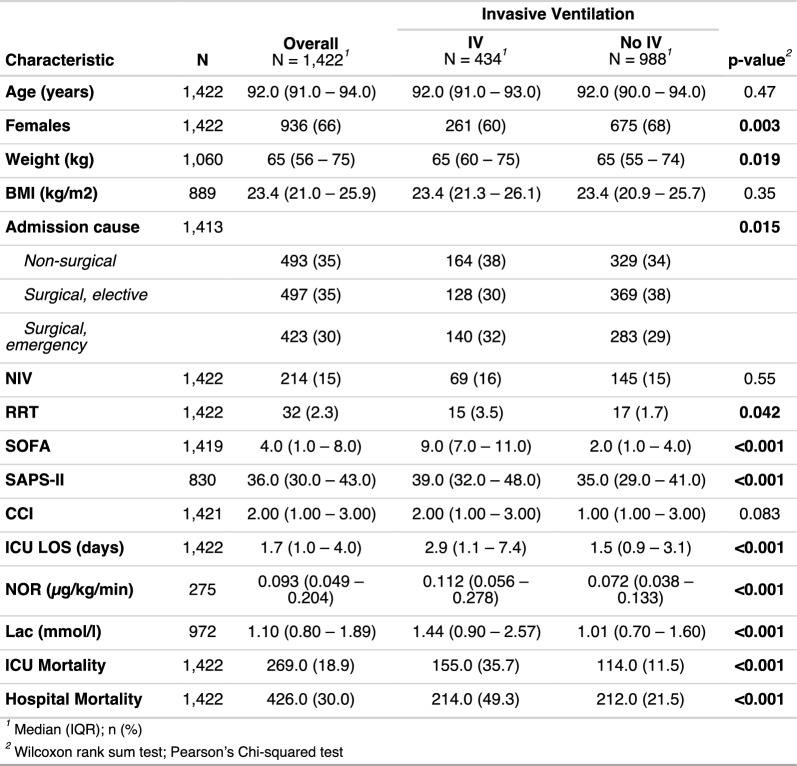
Baseline characteristics of ICU patients aged ≥ 90 years, stratified by invasive ventilation (IV) status

*IV* invasive ventilation; *NIV* non-invasive ventilation; *RRT* renal replacement therapy; *SOFA* Sequential Organ Failure Assessment; *SAPS II* Simplified Acute Physiology Score II; *CCI* Charlson Comorbidity Index; *LOS* length of stay; *NOR* norepinephrine; *Lac* Lactate; *ICU* Intensive care unit

Patients receiving IV had significantly higher illness severity scores (SOFA, SAPS II), ICU length of stay, norepinephrine requirements, and lactate levels compared to those not ventilated. Norepinephrine and lactate values were calculated as time-weighted averages within the first 6 h of admission for each patient. Hospital and ICU mortality were more than twice as high in the IV group

Comorbidities were common across the cohort, with arterial hypertension (72%), atrial fibrillation (41%), and dementia (19%) being the most prevalent. Gastric ulcers were more common in the IV group (6.0% vs. 3.1%, p = 0.012), as was hemiparesis (5.8% vs. 2.3%, p < 0.001). In contrast, aortic stenosis (12.4% vs. 23.5%, p < 0.001) and previous transcatheter aortic valve implantation (TAVI; 5.3% vs. 15.6%, p < 0.001) were more frequently observed in the no-IV group. The overall burden of comorbidity was similar between IV and no-IV groups; median CCI was 2 [IQR 0–3] vs. 1 [IQR 1–3], respectively (p = 0.075). Consult Supplementary Table S1 for further details. Pre-admission residence did not differ between IV and no-IV patients (65% admitted from home, 7% from assisted living, 28% from long-term nursing homes; global p = 0.61). Among hospital survivors (n = 996), 45% were discharged home, 9.4% to short-term nursing or rehabilitation, 30% to long-term nursing homes, and 16% had an unknown destination. Compared with non-ventilated patients, those receiving IV were less often discharged home (38% vs. 47%, p = 0.021) and more frequently to short-term nursing facilities (14% vs. 8.2%, p = 0.016), while discharge to long-term nursing homes did not differ significantly (27% vs. 30%, p = 0.408).

### Ventilation characteristics

Ventilation characteristics showed that among patients who received IV, the median duration was 13 h (IQR 4–44). Stratification by IV duration revealed that 66% of patients were ventilated for < 24 h, while 16% and 17% were ventilated for 24–72 and > 72 h, respectively. Respiratory rate, minute volume, and PEEP differed significantly between these subgroups (all p < 0.002; see Supplementary Table S2). NIV was administered to 15% (n = 214) of patients, with a median duration of 13 h (IQR 3–54).

In the subgroup of patients not ventilated at ICU admission (n = 1,262), 22% (n = 277) subsequently required IV and 16% (n = 196) received NIV during their ICU stay. Among those requiring IV after ICU admission, ICU and hospital mortality were 34% and 48% (p < 0.001). SOFA, SAPS-II, norepinephrine, and lactate levels were all significantly higher at admission in IV patients, while CCI did not differ significantly (2.0 [IQR:1.0–3.0] vs. 1.0 [IQR:1.0–3.0], p = 0.083). The proportion of female patients was again lower in the IV group (58% vs. 68%, p = 0.001). Consult Supplementary Table S3 for further details.

### Mortality and survival

**Fig. 1:**
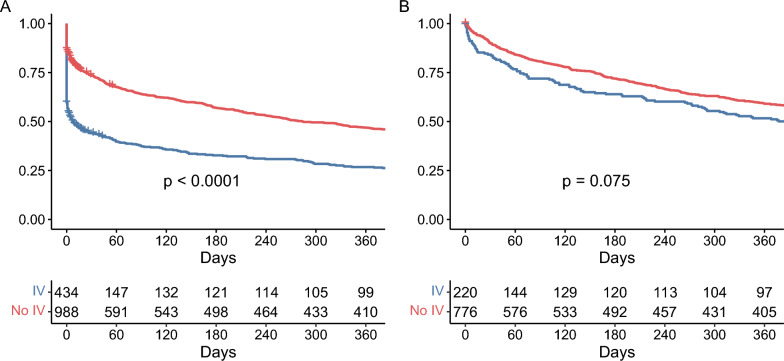
Kaplan–Meier estimates of survival in ICU patients aged ≥ 90 years, stratified by invasive ventilation (IV) status. **1A.** Survival from ICU admission in the full cohort (n = 1422) comparing patients who received IV (blue) to those who did not (red); survival was significantly lower in the IV group (p < 0.0001). **1B.** Survival among hospital survivors only (n = 996), showing a trend towards worse survival for patients that required IV (p = 0.075). *IV* invasive ventilation; *ICU* intensive care unit

ICU mortality in the IV group was 35.7% (n = 155) compared to 11.5% (n = 114) in the no-IV group (p < 0.001), while hospital mortality was 49.3% (n = 214) vs. 21.5% (n = 212; p < 0.001*;* see Table 1)*.* Of those that required IV at any point and died in the ICU (11%, n = 157), 40.7% (n = 64) were ventilated within 6 h before death. Among patients discharged alive from the hospital (n = 996, 70%), 1-year mortality was numerically higher in those who had received IV, but this difference did not reach statistical significance (p = 0.075; see Fig. 1). At 12 months after discharge from the hospital alive, 44.1% (91/220) of patients in the IV group and 36.1% (280/776) in the no-IV group were deceased (p = 0.177), corresponding to an estimated 1-year mortality risks of 48.3% (95% CI 40.6–54.9%) for the IV group and 40.9% (95% CI 37.1–44.5%) for the no-IV group among hospital survivors. Regarding the complete cohort (not just hospital survivors), 1-year mortality risks were 73.2% (95% CI 68.5–77.3%) and 53.4% (95% CI 50.0–56.6%), respectively.

**Fig. 2:**
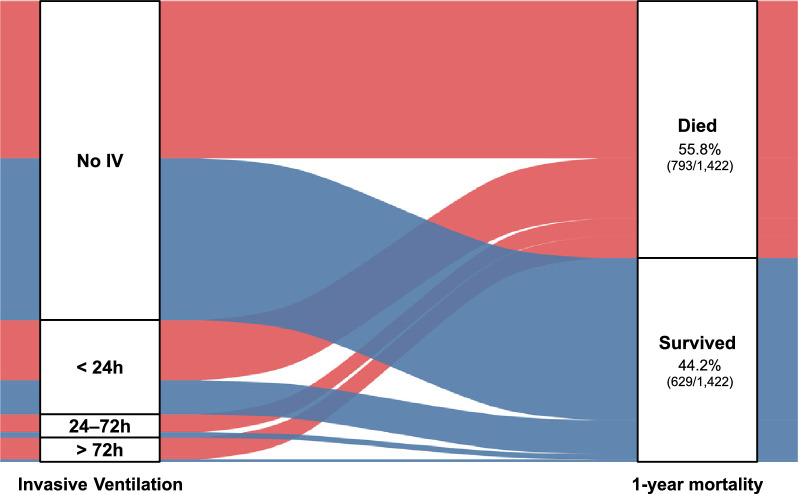
Patient flow from invasive ventilation duration to 1-year mortality in ICU patients aged ≥ 90 years. The Sankey diagram displays the distribution of patients across invasive ventilation categories (no IV, < 24 h, 24–72 h, > 72 h) and 1-year vital status (died, survived). Overall, 55.8% (793/1422) of patients died within 1 year. One-year mortality by ventilation category was 49.3% (485/984) without invasive ventilation, 63.9% (186/291) after < 24 h of ventilation, 76.4% (55/72) after 24–72 h, and 89.3% (67/75) after > 72 h. *ICU* intensive care unit, *IV* invasive ventilation

Patients ventilated for more than 72 h (5.27%, n = 75) had the poorest survival trajectories, while those ventilated for shorter durations showed outcomes more *comparable to non-ventilated patients (*see Supplementary Figure S1 for Kaplan–Meier curves and Fig. 2 for the corresponding Sankey diagram of patient flow from ventilation duration groups through to 1-year survival status).

**Hospital mortality (multivariable logistic regression).** Multivariable logistic regression identified non-surgical admission (OR 2.68, 95% CI 1.74–4.16, p < 0.001), SOFA scores ≥ 8 (OR 3.46, 95% CI 2.12–5.69, p < 0.001), CCI ≥ 3 (OR 1.74, 95% CI 1.08–2.83, p = 0.023), IV duration > 72 h (OR 4.01, 95% CI 1.92–8.70, p < 0.001), and lactate ≥ 4 mmol/l (OR 6.67, 95% CI 4.26–10.55, p < 0.001) as independent predictors of hospital mortality (Table 2).

**Table 2:**
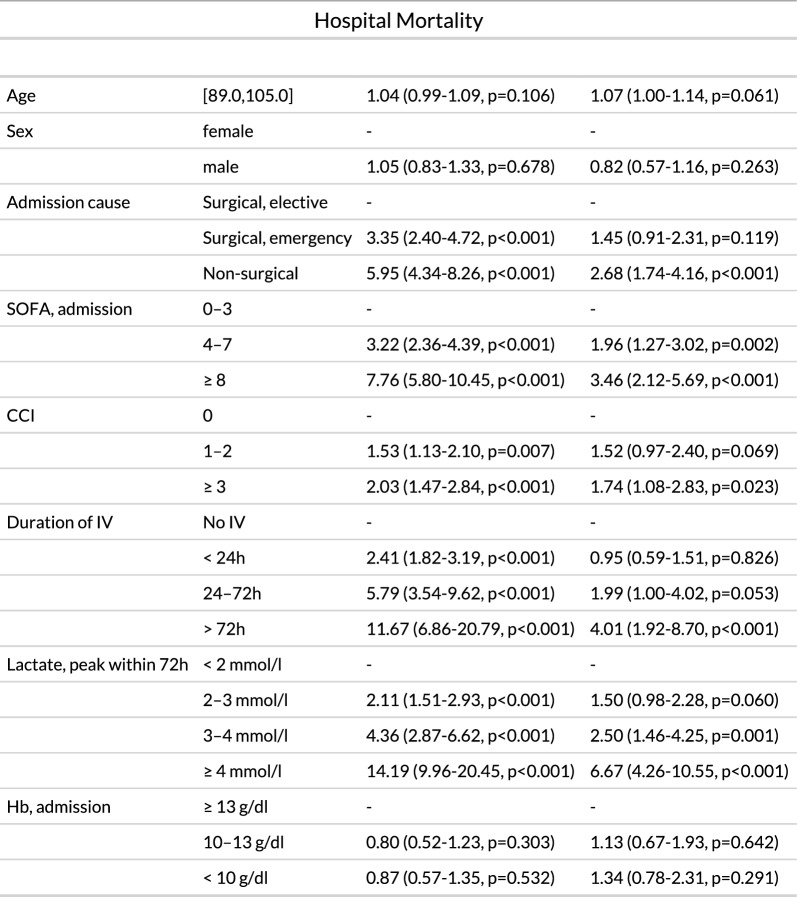
Multivariable logistic regression model for hospital mortality in ICU patients aged ≥ 90 years

*IV* Invasive ventilation; *SOFA* Sequential Organ Failure Assessment; *CCI* Charlson Comorbidity Index; *Hb* Haemoglobin; *OR* Odds ratio

The analysis identified prolonged invasive ventilation (> 72 h), elevated lactate (≥ 4 mmol/l), non-surgical admission, high SOFA scores (≥ 8), and high comorbidity burden (CCI ≥ 3) as independent predictors of in-hospital death. Odds ratios (ORs) with 95% confidence intervals are shown for both univariate and multivariable analyses

**Long-term mortality among hospital survivors (Cox regression).** For patients discharged alive from the hospital, multivariable Cox regression (n = 580; 513 events) showed that higher age (HR [hazard ratio] 1.08 per year, p < 0.001), non-surgical admission (HR 1.60, p < 0.001), CCI ≥ 3 (HR 1.52, p = 0.001), and haemoglobin < 10 g/dl at admission (HR 1.63, p = 0.002) were independently associated with higher follow-up mortality (Supplementary Table S4)*.* The model demonstrated moderate discrimination (c-index 0.618), although the proportional hazards assumption was not met due to time-dependent effects of admission cause, SOFA, ventilation duration, and lactate.

## Discussion

In this retrospective cohort of 1,422 ICU patients aged ≥ 90 years were included. IV was administered in 31% (n = 434), being associated with a significantly higher ICU and hospital mortality (35.7% vs. 11.5% and 49.3% vs. 21.5%, both p < 0.001). In multivariable analysis, IV lasting 24–72 h showed a non-significant trend towards higher hospital mortality (OR 1.99; p = 0.053), whereas IV > 72 h was significantly associated with increased mortality (OR 4.01; p < 0.001). Among patients discharged alive from the hospital, 1-year mortality was numerically higher in those who had received invasive ventilation, but this difference did not reach statistical significance (p = 0.075) and should be interpreted cautiously. Given the observational design and the absence of key geriatric variables, these findings should be interpreted as indicating that patients who become sick enough to require (and remain on) invasive ventilation constitute a particularly high-risk subgroup, rather than proving a causal effect of invasive ventilation itself on mortality.

Our findings underscore the need for informed decision-making, including early goals-of-care discussions and consideration of time-limited ICU trials in this vulnerable population, with routine involvement of palliative care and geriatric teams when invasive ventilation is being considered and explicit scrutiny of whether escalation is consistent with the patient’s values and expected quality of life.

### Invasive ventilation and mortality

Our findings align with and extend existing evidence on outcomes of IV in very old ICU patients, particularly nonagenarians. Le Borgne et al. reported ICU and hospital mortality rates of 35.7% and 42.6%, respectively, in nonagenarians–higher than in our overall cohort (18.9% and 30.0%). However, their cohort had a higher proportion of IV (49.2% vs. 30.5%), likely contributing to the observed differences. While ventilation-stratified outcomes were not reported, IV emerged as the strongest independent predictor of ICU mortality (OR 4.83). Among patients who died in the ICU, length of stay was similar between IV and no-IV groups (median 2.6 vs. 2.5 days, p = 0.31) [26]. In our cohort, overall ICU stays were generally short (median 1.7 days), and two-thirds of ventilated patients received IV for less than 24 h, consistent with a case mix that includes many brief stabilisation or peri-procedural episodes rather than prolonged respiratory failure; this limits generalisability to settings with longer, more protracted ICU courses and sustained organ support.

Larger national and administrative datasets provide a complementary perspective on outcomes of very old, ventilated patients. Karagiannidis et al. reported a 59.0% mortality among ventilated patients ≥ 80 years in German administrative data. However, as their analysis included both ICU and non-ICU settings and did not separately analyse nonagenarians, comparisons to our cohort are limited [15]. Haas et al. analysed Dutch national data comparing octogenarians and nonagenarians. Of 104,754 ICU patients aged ≥ 80 years, 9495 (9%) were ≥ 90 years. ICU mortality was slightly lower in nonagenarians (13.8% vs. 16.1%; p < 0.001), and hospital mortality was similar (26.1% vs. 25.7%; p = 0.41). IV within 24 h was required in 23.8% of nonagenarians, though later initiation was not assessed [27]. While the overall 1-year mortality in our cohort (55.7%) approximates the mortality reported by Haas et al. (55%), the notably higher mortality in ventilated patients (69.8% vs. 49.5% at 1 year) underscores the importance of accounting for timing and duration of IV. When interpreting 1-year mortality, it is essential to consider the expected background risk in the general population. According to official German life tables, the probability of death within 1 year for 92-year-old individuals (median age of our cohort) is 23.8% for men and 19.3% for women [3].

These findings highlight the resource-intensive nature of IV in nonagenarians, often without long-term survival benefit, and underscore the need for early goal-directed decision-making to avoid prolonged non-beneficial ICU stays.

Further stratification by admission category and duration of IV revealed distinct patterns of 1-year mortality*.* Among patients admitted for non-surgical reasons, mortality was consistently high across all ventilation durations, exceeding 85% in those ventilated for any period, and reaching 63.2% even among those not receiving IV. In contrast, surgical admissions showed greater heterogeneity. Elective surgical patients had the most favourable outcomes, particularly those not ventilated (35.0% mortality) or ventilated for < 24 h (41.3%). However, prolonged ventilation (> 72 h) in any group—elective, emergency, or non-surgical—was associated with uniformly poor prognosis, with 1-year mortality exceeding 85% in all cases. These findings highlight the combined impact of admission cause and ventilation burden on long-term survival, and underscore the poor prognosis associated with prolonged IV in this age group, regardless of admission context.

Pre-admission residence and discharge destination provide some indirect context regarding vulnerability and recovery. Most patients were admitted from home, yet only 45% of hospital survivors were discharged home, with the remainder transferred to short- or long-term nursing or rehabilitation facilities. This pattern suggests that many nonagenarians did not return to independent living after critical illness, even when surviving to hospital discharge, but these descriptors remain crude and cannot replace formal frailty or functional assessments.

Peak lactate concentration within the first 72 h of ICU admission was one of the strongest independent predictors of hospital mortality in our cohort. A peak lactate ≥ 4 mmol/l was associated with more than sixfold increased odds of hospital mortality (OR 6.67, 95% CI 4.26–10.55, p < 0.001), and even moderate elevations between 3 and 4 mmol/l carried a significantly higher risk (OR 2.50, 95% CI 1.46–4.25, p = 0.001; see Table 2). In the absence of robust trial data to guide ICU management in very old patients, such routinely available markers offer valuable support for clinical decision-making. Elevated lactate–especially when rising above 3 mmol/l–may help clinicians identify patients at risk of poor outcomes early in the ICU stay and inform timely goals-of-care discussions.

### Ethical considerations

The treatment of nonagenarians in the ICU raises complex ethical questions, particularly considering high mortality rates, limited long-term survival, and the increasing strain on critical care resources. Even when ICU survival is achieved, long-term follow-up studies in ARDS and general critical illness have shown substantial and persistent limitations in physical function, cognition, mental health, and return to work, with markedly reduced health-related quality of life [18–21]. In this retrospective dataset, we could not reliably capture advance directives, formal palliative care consultations, or the timing and content of goals-of-care discussions, and we are therefore unable to quantify how these factors influenced decisions to initiate or forgo invasive ventilation. This part of the Discussion should thus be understood as an interpretation of our outcome data in the context of the existing literature, rather than as a description of measured decision-making processes in this cohort.

Initiating conversations about therapy limitations for the first time during an ICU stay risks being reactive rather than anticipatory, thus, compromising the quality of end-of-life care. In practice, these discussions are often lacking: In one study only 12.7% of elderly patients had been asked about their willingness to be admitted to an ICU [28]. In others, treatment preferences were frequently unknown or reported mainly by surrogates rather than by patients themselves [10, 29].

Against this background, decisions to withhold or withdraw life-sustaining treatments should be based on transparent, multidisciplinary discussions that consider prognosis, values, and expected quality of life. For nonagenarians being considered for invasive ventilation, this argues for early palliative care input, structured advance care planning, and explicit discussion of non-invasive, comfort-focused options should be standard rather than exceptional.

Time-limited ICU trials, defined as a pre-agreed period of intensive treatment with explicit criteria for reassessment and potential de-escalation or withdrawal if there is no meaningful improvement, may offer a pragmatic framework for managing uncertainty in this setting [30].

## Limitations

This study has several limitations. Its retrospective, single-centre design may limit generalisability to ICUs with different case mixes, admission criteria, or treatment approaches. Selection bias is inherent, as patients admitted to the ICU likely represent a group likely already considered suitable for aggressive care, potentially excluding frailer individuals or those with established limitations of therapy, and we were unable to capture patients who were not referred to the ICU or in whom invasive ventilation was withheld or declined; our findings therefore apply only to this selected population. Moreover, ICU stays were typically short (median 1.7 days), and many episodes of invasive ventilation were brief, complicating interpretation of the impact of IV and limiting comparability with cohorts characterised by longer ICU courses and prolonged organ support.

Additionally, key geriatric parameters—such as frailty, pre-ICU functional status, cognitive impairment, and patient preferences—were not available, limiting the ability to assess the broader appropriateness and outcomes of ICU care in this age group; pre-admission residence and discharge destination can only be interpreted as rough proxies rather than formal frailty measures. Decisions to withhold or withdraw life-sustaining treatments, the presence or absence of advance directives, formal palliative care involvement, and the timing or content of goals-of-care discussions were not systematically documented, precluding an analysis of how patient preferences and end-of-life decisions influenced survival; the ethical considerations discussed are therefore interpretative rather than directly supported by preference data from this cohort. Long-term survival analyses were restricted to hospital survivors, so 1-year mortality estimates are conditional on discharge alive and subject to survivorship bias when applied to all ICU nonagenarians. Lastly, while long-term survival was captured through population registry data, post-discharge outcomes such as quality of life, functional recovery, and readmissions were not assessed—factors that are essential for understanding the true benefit of intensive care in nonagenarians.

## Conclusion

Invasive ventilation in ICU patients aged ≥ 90 years was strongly associated with higher ICU and hospital mortality, particularly when prolonged beyond 72 h. Despite comparable comorbidity profiles, ventilated patients presented with greater illness severity and worse trajectories. Simple, routinely available markers—such as elevated lactate, high SOFA scores, and prolonged ventilation—may help identify patients at risk of poor outcomes. Given the observational design and residual confounding by illness severity, these associations should not be interpreted as proof that invasive ventilation itself causes excess mortality. As the number of very old ICU patients continues to rise, these findings underscore the need for early, structured, and patient-centred decisions around the use of invasive ventilation in this vulnerable population.

## Supplementary Information

Additional file 1.

## Data Availability

The datasets analysed during the current study are not publicly available due to institutional data protection regulations but are available from the corresponding author on reasonable request and with permission from the University Medical Center Hamburg-Eppendorf.

## Abbreviations

CCI: Charlson Comorbidity Index
CI: Confidence Interval
HR: Hazard Ratio
ICM: Integrated Care Manager (Draeger)
ICU: Intensive Care Unit
IV: Invasive Ventilation
LOS: Length of Stay
NIV: Non-Invasive Ventilation
OR: Odds Ratio
RRT: Renal Replacement Therapy
SAPS II: Simplified Acute Physiology Score II
SOFA: Sequential Organ Failure Assessment
STROBE: Strengthening the Reporting of Observational Studies in Epidemiology
TLT: Time-Limited Trial
