# Supplementary material for: Invasive ventilation and mortality in critically ill nonagenarians: a retrospective cohort study
Source: Eur J Med Res. 2026 Jan 28;31:337. doi: 10.1186/s40001-026-03928-6 (PMC12924332; doi:10.1186/s40001-026-03928-6)
Supplement: Supplementary file 1 — Additional file 1. [file 40001_2026_3928_MOESM1_ESM.docx]

# Supplement

## Supplementary Table 1


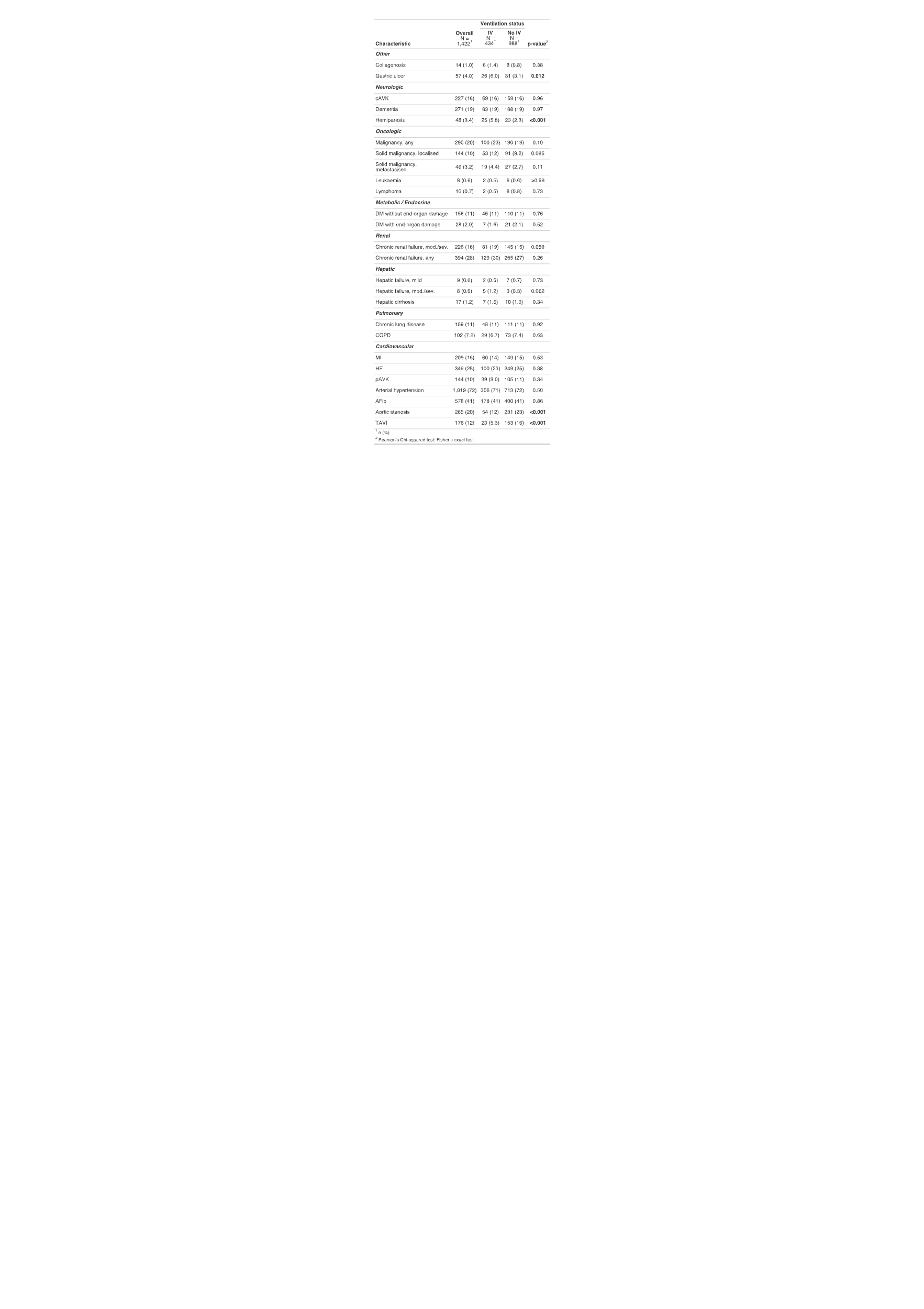
**Supplementary Table 1**.
Prevalence of selected comorbidities stratified by invasive ventilation (IV) status among ICU patients aged ≥90 years. Most comorbid conditions were similarly distributed between IV and no-IV groups. However, hemiparesis (5.8% vs. 2.3%, p < 0.001), aortic stenosis (12% vs. 23%, p < 0.001), and history of transcatheter aortic valve implantation (TAVI; 5.3% vs. 16%, p < 0.001) were significantly less frequent in the IV group. Gastric ulcer was also more prevalent among IV patients (6.0% vs. 3.1%, p = 0.012). These differences may reflect selection factors influencing decisions around ventilation.

**Abbreviations:** AFib – atrial fibrillation, CCI – Charlson Comorbidity Index, COPD – chronic obstructive pulmonary disease, DM – diabetes mellitus, HF – heart failure, ICU – intensive care unit, IV – invasive ventilation, MI – myocardial infarction, mod./sev. – moderate or severe, pAVK – peripheral arterial occlusive disease, TAVI – transcatheter aortic valve implantation.

## Supplementary Table 2


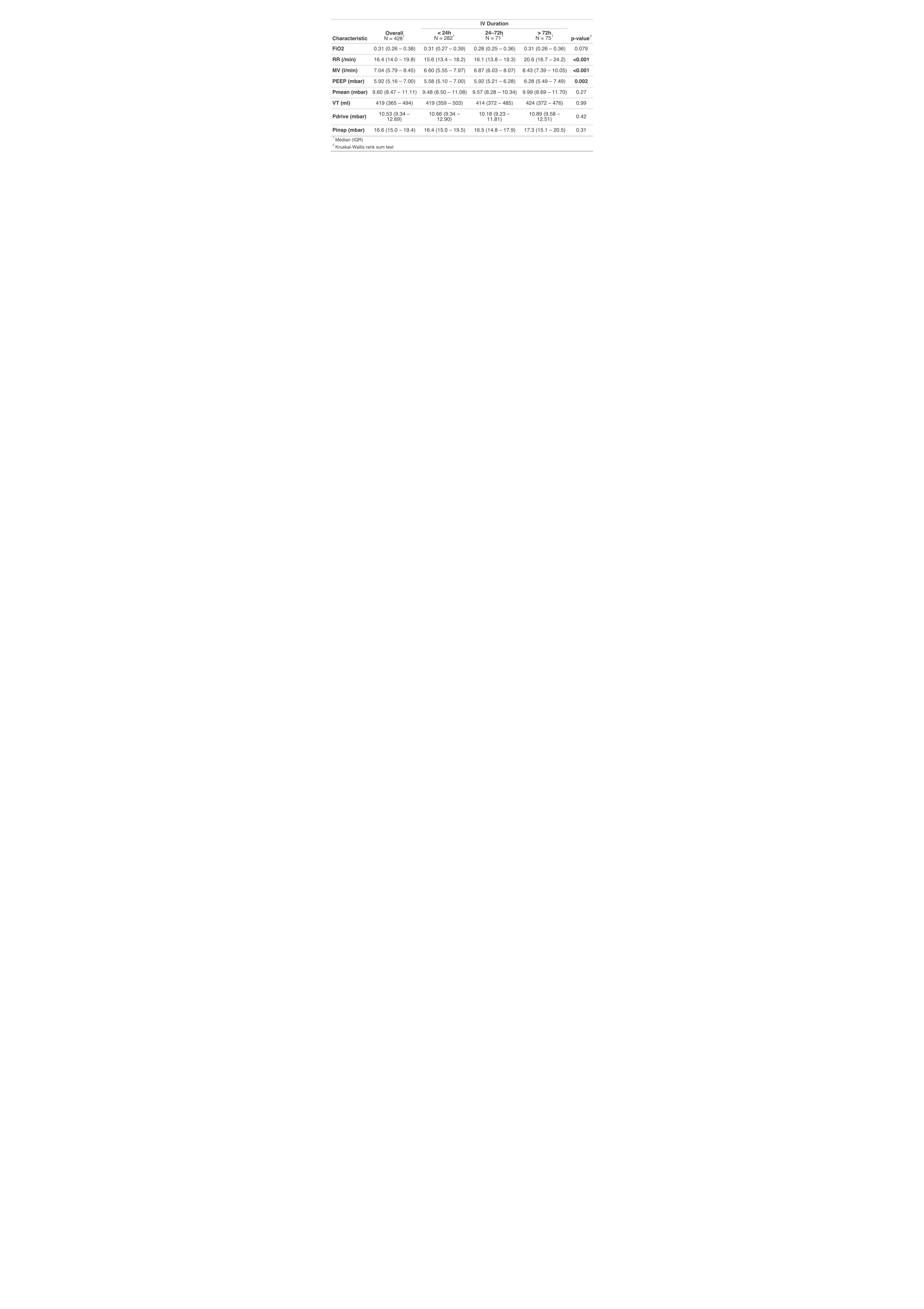


**Supplementary Table 2.**Ventilator settings stratified by duration of IV. Patients ventilated for >72 hours had significantly higher respiratory rates (median 20.6/min), minute volumes (8.43 l/min), and positive end-expiratory pressure (PEEP; 6.28 mbar) compared to those ventilated for shorter periods (<24h or 24–72h), with p < 0.001, < 0.001, and = 0.002, respectively. No significant differences were observed for tidal volume, mean airway pressure (Pmean), inspiratory pressure (Pinsp), or driving pressure (Pdrive) across duration groups.

**Abbreviations:** FiO2 – fraction of inspired oxygen, RR – respiratory rate, MV – minute volume, PEEP – positive end-expiratory pressure, Pmean – mean airway pressure, VT – tidal volume, Pdrive – driving pressure, Pinsp – inspiratory pressure, IQR – interquartile range, IV – invasive ventilation.

## Supplementary Table 3


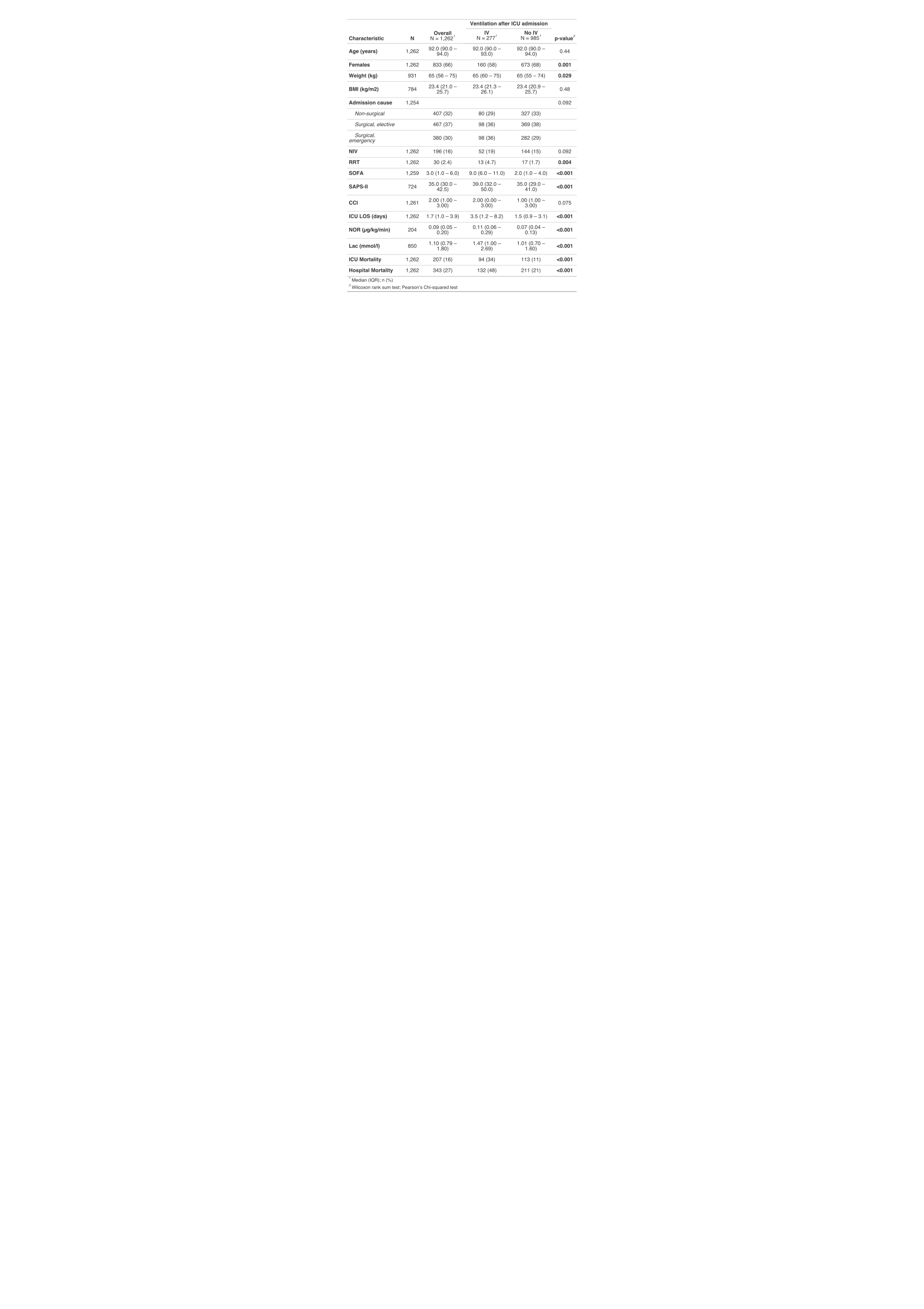


**Supplementary Table 3.**Baseline characteristics and outcomes in patients not invasively ventilated at ICU admission, stratified by whether IV was subsequently initiated during the ICU stay. Patients requiring delayed IV (n = 277) had significantly higher illness severity at admission, including elevated SOFA (median 9.0 vs. 2.0), SAPS-II (39.0 vs. 35.0), norepinephrine requirements (0.11 vs. 0.07 µg/kg/min), and lactate levels (1.47 vs. 1.01 mmol/l), all p < 0.001. ICU and hospital mortality were markedly higher in this group (34% vs. 11% and 48% vs. 21%, respectively; both p < 0.001), despite similar comorbidity burden (CCI) and age. ICU length of stay was also significantly prolonged (3.5 vs. 1.5 days, p < 0.001).

**Abbreviations:** BMI – body mass index, CCI – Charlson Comorbidity Index, ICU – intensive care unit, IV – invasive ventilation, LOS – length of stay, Lac – lactate, NIV – non-invasive ventilation, NOR – norepinephrine, RRT – renal replacement therapy, SAPS-II – Simplified Acute Physiology Score II, SOFA – Sequential Organ Failure Assessment.

## Supplementary Table 4


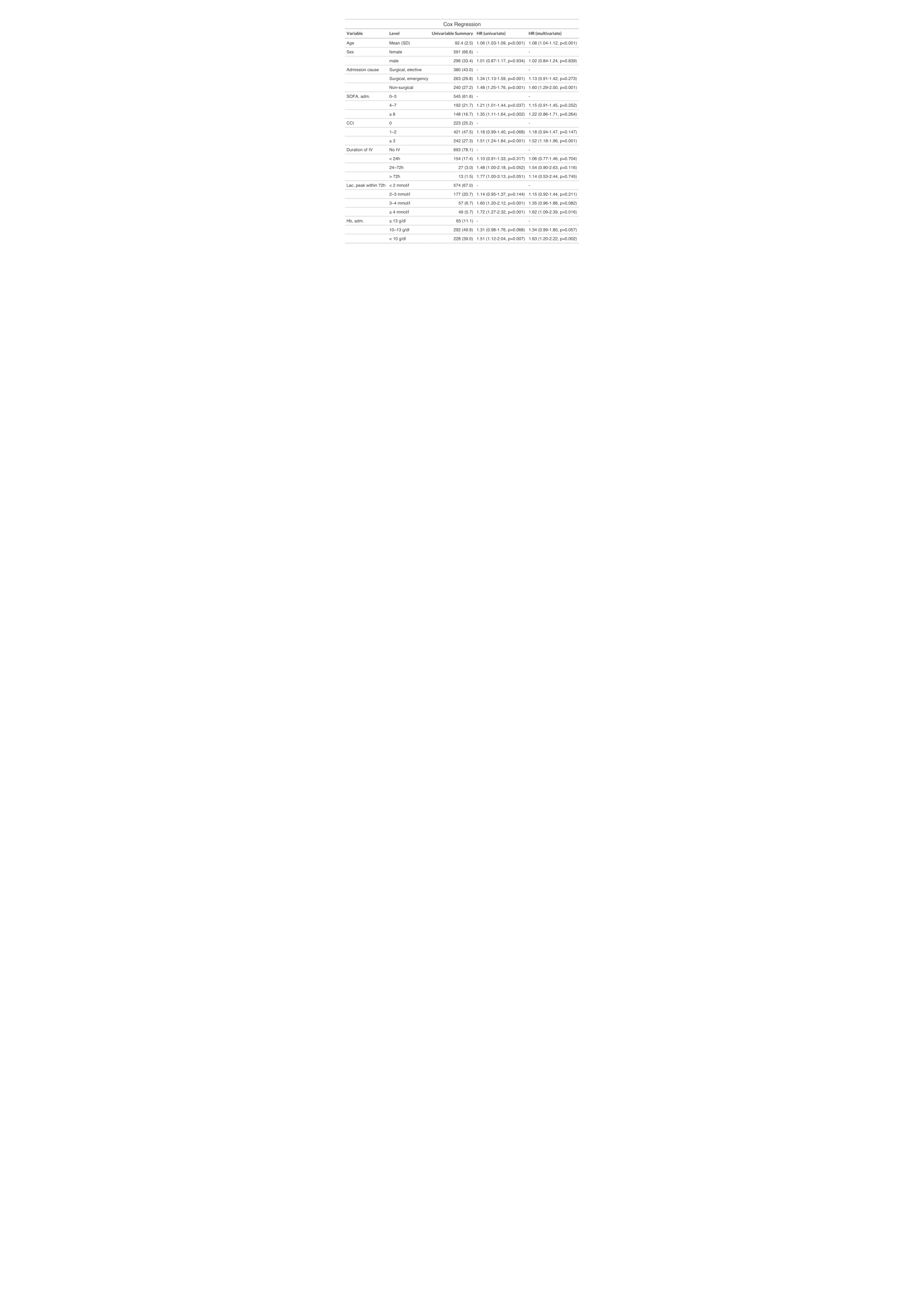


**Supplementary Table 4.**
Cox regression model for all-cause mortality over the full follow-up period in patients discharged from the hospital alive. In the multivariable analysis, significant independent predictors of increased mortality included older age (HR 1.08 per year), non-surgical admission (HR 1.60), CCI ≥3 (HR 1.52), peak lactate ≥ 4 mmol/l within 72 hours (HR 1.62, p < 0.001), and haemoglobin <10 g/dl (HR 1.63, p = 0.002).

**Abbreviations:** CCI – Charlson Comorbidity Index, Hb – haemoglobin, HR – hazard ratio, ICU – intensive care unit, IV – invasive ventilation, Lac – lactate, SOFA – Sequential Organ Failure Assessment.

## Supplementary Figure 1


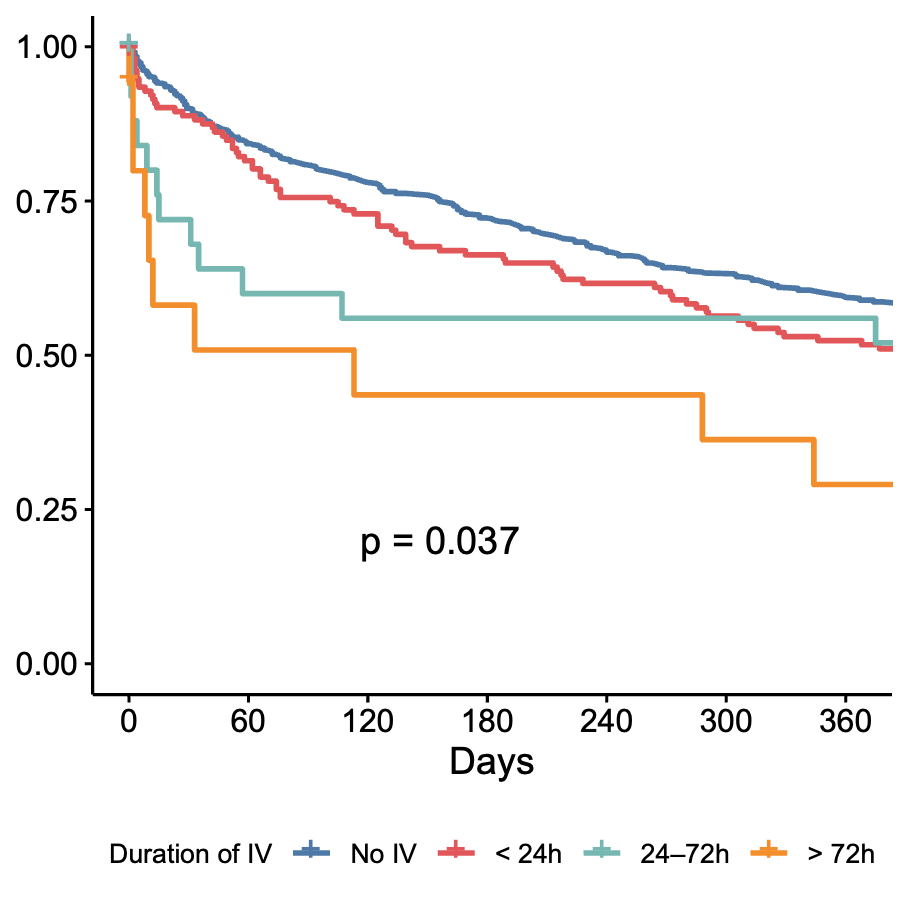


**Supplementary Figure 1.**Kaplan–Meier survival estimates for patients discharged from hospital alive, stratified by duration of invasive ventilation (IV). Patients ventilated for more than 72 hours showed the poorest post-discharge survival, with significantly lower one-year survival compared to all other groups (p = 0.037). Shorter durations of IV (<24 h and 24–72 h) were associated with intermediate outcomes, while patients not receiving IV had the most favourable long-term survival.

**Abbreviations:** IV – invasive ventilation.
